# Redundancy and metabolic function of the glutamine synthetase gene family in poplar

**DOI:** 10.1186/s12870-014-0365-5

**Published:** 2015-01-22

**Authors:** Vanessa Castro-Rodríguez, Angel García-Gutiérrez, Rafael A Cañas, Ma Belén Pascual, Concepción Avila, Francisco M Cánovas

**Affiliations:** Departamento de Biología Molecular y Bioquímica, Facultad de Ciencias, Campus Universitario de Teatinos, Universidad de Málaga, 29071 Málaga, Spain

**Keywords:** *Populus*, Gene family, Gene duplication, Glutamine, Ammonium assimilation

## Abstract

**Background:**

Glutamine synthetase (GS; EC: 6.3.1.2, L-glutamate: ammonia ligase ADP-forming) is a key enzyme in ammonium assimilation and metabolism in higher plants. In poplar, the GS family is organized in 4 groups of duplicated genes, 3 of which code for cytosolic GS isoforms (GS1.1, GS1.2 and GS1.3) and one group that codes for the choroplastic GS isoform (GS2). Our previous work suggested that GS duplicates may have been retained to increase the amount of enzyme in a particular cell type.

**Results:**

The current study was conducted to test this hypothesis by developing a more comprehensive understanding of the molecular and biochemical characteristics of the poplar GS isoenzymes and by determinating their kinetic parameters. To obtain further insights into the function of the poplar GS genes, *in situ* hybridization and laser capture microdissections were conducted in different tissues, and the precise GS gene spatial expression patterns were determined in specific cell/tissue types of the leaves, stems and roots. The molecular and functional analysis of the poplar GS family and the precise localization of the corresponding mRNA in different cell types strongly suggest that the GS isoforms play non-redundant roles in poplar tree biology. Furthermore, our results support the proposal that a function of the duplicated genes in specific cell/tissue types is to increase the abundance of the enzymes.

**Conclusion:**

Taken together, our results reveal that there is no redundancy in the poplar GS family at the whole plant level but it exists in specific cell types where the two duplicated genes are expressed and their gene expression products have similar metabolic roles. Gene redundancy may contribute to the homeostasis of nitrogen metabolism in functions associated with changes in environmental conditions and developmental stages.

**Electronic supplementary material:**

The online version of this article (doi:10.1186/s12870-014-0365-5) contains supplementary material, which is available to authorized users.

## Background

Woody plants constitute one of the most important economic and ecological resources on Earth. The forest ecosystems play an important role in the production of the world’s biomass. Therefore, they are a necessary factor that must be considered when addressing climate change and the maintenance of biological diversity. Trees are an inestimable resource in various industries such as wood, pulp, paper, biofuel and other useful material of commercial importance [1]. The molecular biology of trees is a field that is experiencing extraordinary advances especially because different genomic and transcriptomic projects are providing a huge amount of valuable information to understand the molecular basis underlying the physiological regulation of gene expression.

Nitrogen metabolism is a fundamental area of research in plant biology. Nitrogen, a constitutive element of amino acids and nucleotides, is a limiting factor in the growth and development of land plants and constitutes a true challenge for their survival [2]. Terrestrial plants have evolved metabolic pathways to assimilate and distribute nitrogen for the biosynthesis of a wide range of molecules. Nitrogen is both essential and limiting, and plants have developed systems to guarantee its economy such as the glutamine synthetase (GS)/glutamate synthase (GOGAT) cycle [3]. The enzyme GS (EC: 6.3.1.2) synthesizes glutamine incorporating ammonium to glutamate in the presence of ATP, while GOGAT (EC: 1.4.7.1) generates glutamate by transferring the amide group of glutamine to α-ketoglutarate. The amino acids glutamine and glutamate are the main nitrogen donors for the biosynthesis of a wide variety of nitrogenous compounds. X-ray crystallography of maize ZmGS1a [4] and Medicago MtGS1a [5] demonstrated that plant GS is a decameric enzyme. The protein is composed of two face-to-face pentameric rings with active sites located at the interfaces between the N-terminal and C-terminal domains of two neighboring subunits within a pentameric ring, which results in a total of 10 active sites per GS decamer [4].

Plants have also developed systems for ammonium reassimilation from secondary sources to avoid losing biological nitrogen. During photorespiration the mitochondrial decarboxylation of glycine generate important quantities of ammonium, which are then incorporated to carbon skeletons in the chloroplast through the GS/GOGAT cycle [6]. These metabolic activities are combined because of the strict spatial association of mitochondria, peroxisome and chloroplast, and because they prevent the toxic accumulation of ammonium and nitrogen loss [7]. Even though lignin, a polymeric compound especially important in woody plants, does not contain nitrogen, phenylalanine metabolism is required to channel photosynthesis-derived carbon to phenylpropanoid biosynthesis. The ammonium released in the reaction catalyzed by phenylalanine ammonia lyase is recycled by the GS/GOGAT cycle, which allows it to be reincorporated into the continuous synthesis of phenylalanine, and consequently lignin and other phenolic compounds [8,9]. Furthermore, GS is also expressed in different physiological situations such as pathogen attack [10,11] or senescence [12].

In plants, cytosolic (GS1) and chloroplastic (GS2) glutamine synthetase isoenzymes have been identified and are found in different intracellular locations that are related to their specialized roles. The chloroplastic GS2 is coded by a single gene in most plant species and has been detected in photosynthetic tissues where it assimilates the ammonium released from photorespiration or nitrate/nitrite reduction [13]. Conversely, GS1 is coded by a small gene family which varies in number among species, and the different isoenzymes are found in different types of cells and tissues according to their different physiological functions [14]. GS1 is mainly found in heterotrophic organs such as roots, seeds, stems, nodules, flowers and fruits, where it assimilates the ammonium from the soil, lignin biosynthesis, stress and senescence [15].

In a previous study [16], it was reported that the GS gene family in poplar is organized into 4 groups of duplicated genes, 3 of which code for cytosolic GS isoforms (GS1.1, GS1.2 and GS1.3) and 1 that codes for the chloroplastic GS isoform (GS2). Our previous findings suggested that the GS duplicates may have been retained to increase the amount of enzyme in particular cell types.

The aim of the current study was to develop a more comprehensive understanding of the molecular structure, biochemical properties, and kinetic parameters of GS isoenzymes and to evaluate the cell- and tissue-specific spatial expression of the individual members of the GS gene family in poplar. The molecular and functional analysis of the GS family and the precise locations of the corresponding mRNA strongly support that GS isoforms play non-redundant roles in poplar tree biology. Our results also support the proposal that the function of the duplicated genes in specific cell types is to increase the abundance of the enzymes. Therefore, while there is no redundancy in the poplar GS family at the plant level, redundancy does exist in specific cell types that express two duplicated genes. This gene redundancy may contribute to maintaining the homeostasis of nitrogen metabolism during processes associated with the changes in glutamine use in multiple metabolic pathways.

## Results

### Expression of active poplar GS isoforms

**Figure 1:**
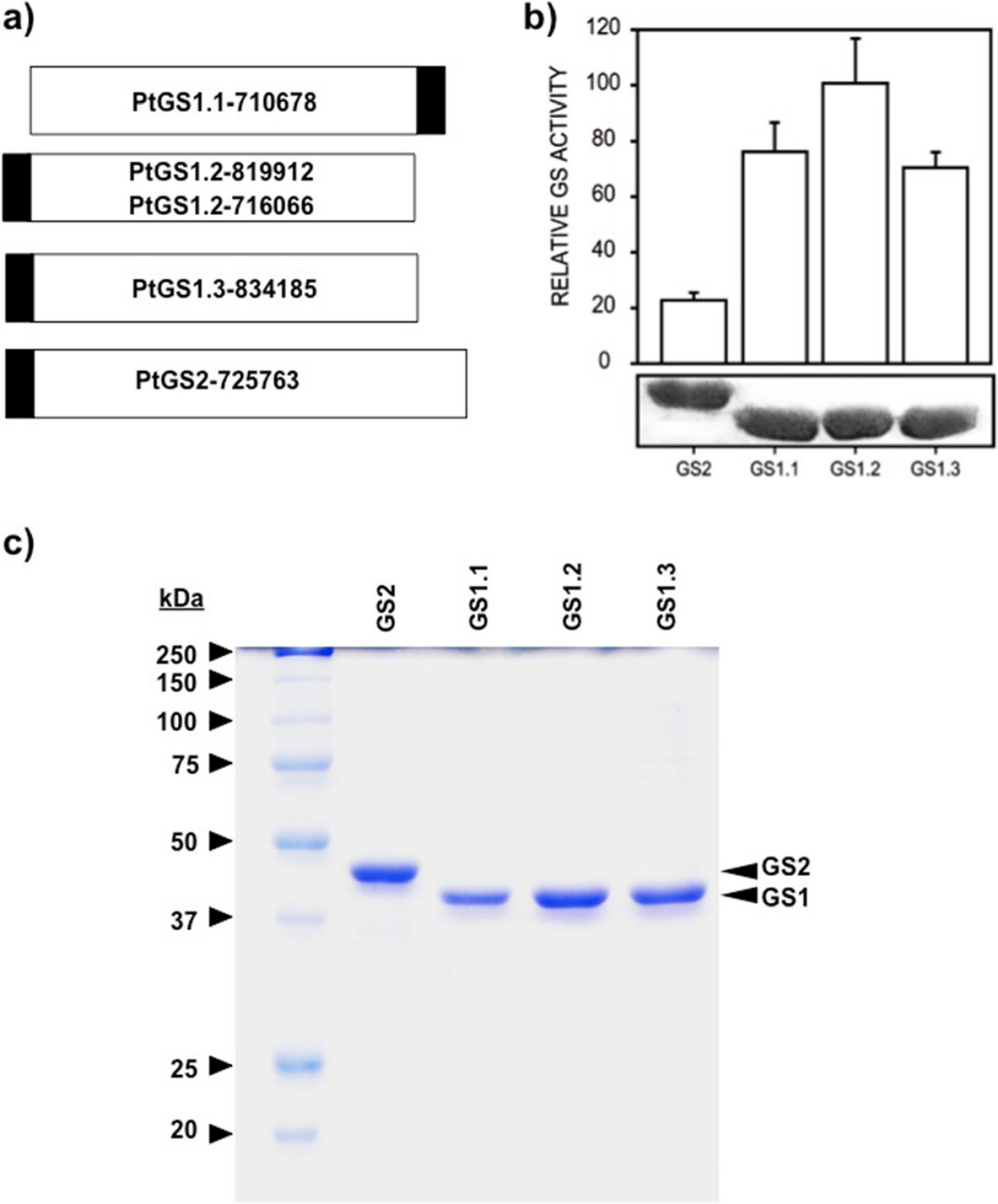
**Recombinant overproduction of poplar glutamine synthetases. a)** Schematic representation of constructs made with *Populus trichocharpa* FLcDNA from N- (left) to C-(right) terminals. White rectangle: FLcDNA; black rectangle: His-tag. **b)** Expression of poplar GS2, GS1.1, GS1.2 and GS1.3 isoenzymes in *E. coli*. Levels of GS activity for each isoenzyme are shown in the upper part of the Figure. The maximum level (100%) of activity was 67 nkatals. Values are the mean ± SD of at least three independent determinations. Immunoblot of the same protein extracts is shown in the lower part of the Figure. **c)** Electrophoretic analysis of the homogenous preparations of poplar GS2, GS1.1, GS1.2 and GS1.3 isoenzymes. Molecular markers were loaded on the left.

The poplar genome contains 4 groups of duplicated genes of GS named *GS1.1, GS1.2, GS1.3* and *GS2,* which are expressed in different organs of the tree [16]. Total intact RNA was isolated from *Populus trichocarpa* clone INRA 101–74 and full-length cDNA (FLcDNA) representatives of GS genes were isolated by RT-PCR using specific primers (see the Methods section for further information). The identity of GS cDNAs was confirmed by sequencing analysis and the corresponding data are presented in Additional file 1. Constructs of His-tag fusion proteins for GS1.1 (PtGS1.1-710678), GS1.2 (PtGS1.2-819912, PtGS1.2-716066), GS1.3 (PtGS1.3-834185), GS2 (PtGS2-725763) were expressed in *Escherichia coli* (Additional file 2: Table S1 and Figure 1a). All poplar GS isoforms were active in bacteria and the specific activities observed varied among the different isoforms, with higher values for GS1 isoforms than for GS2 (Figure 1b, upper panel). The western blot analysis of the bacterial protein extracts demonstrated a parallel accumulation of plant GS polypeptides (Figure 1b, lower panel). Poplar GS1.1, GS1.2, GS1.3 and GS2 holoenzymes were purified to homogeneity by affinity chromatography (Figure 1c). These highly purified enzyme preparations were used for molecular and kinetic analysis.

### Molecular size of poplar GS isoforms

The molecular sizes of the poplar GS polypeptides are shown in Table 1. The predicted values derived from the poplar genome sequence (JGI) were compared with the values derived from cDNA sequencing of the individual FLcDNA (PtGS1.1-710678, PtGS1.2-819912/PtGS1.2-716066, PtGS1.3-834185 and PtGS2-725763) and with the experimental values determined by mass spectrometry analysis of purified preparations of GS1.1, GS1.2, GS1.3 and GS2 recombinant proteins (MS/MALDI). The sizes of the GS1 (39–40 kDa) and GS2 (42 kDa) isoforms determined by MS/MALDI were similar to the values predicted based on the cDNA and JGI (Table 1).

**Table 1:** **Molecular sizes of poplar GS polypeptides (kDa)**

**Polypeptide**	**Genome(JGI)**	**cDNA**	**MS/MALDI**
GS2	42.29	42.29	42.17
GS1.1	39.45	39.21	39.97
GS1.2	38.97	38.96	39.98
GS1.3	39.09	39.19	38.95

**Figure 2:**
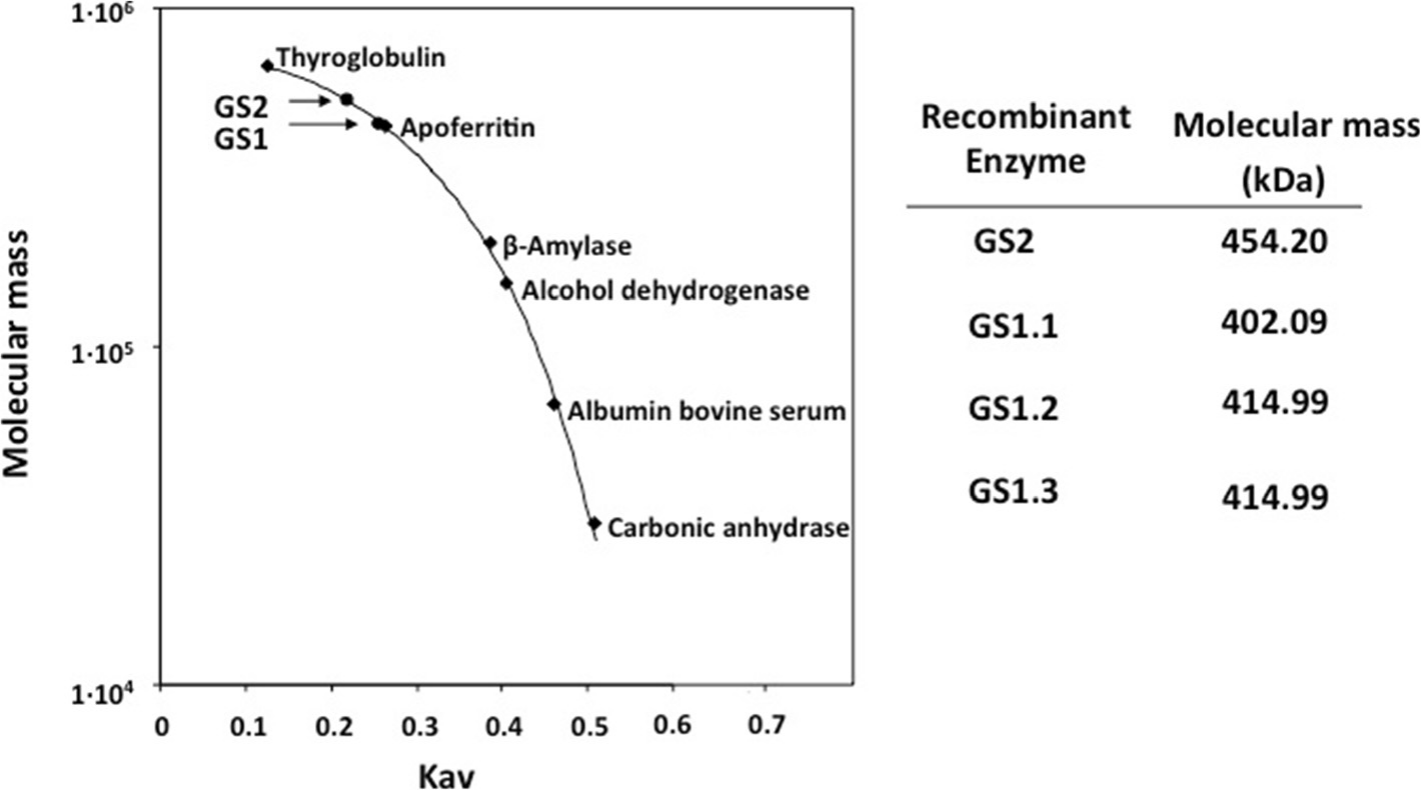
**Molecular mass estimation of poplar GS holoenzymes.** Samples (100 μg) of purified GS2, GS1.1, GS1.2 and GS1.3 native isoenzymes were subjected to Sephacryl S-300 chromatography. The molecular masses were calculated by comparison of the partition coefficient (K_av_) of the GS isoenzymes with proteins of known molecular size. The protein standars used to calibrate the column were: Thyroglobulin (669 kDa), Apoferritin (443 kDa), β-Amylase (200 kDa), Alcohol Dehydrogenase (150 kDa), Albumin (66 kDa) and Carbonic Anhydrase (29 kDa).

The molecular sizes of the poplar GS holoenzymes were determined by gel filtration chromatography through a calibrated column with proteins standards (Figure 2). The GS2 holoenzyme was observed to be 454 kDa and the estimated sizes of the GS1 holoenzymes ranged from 400 to 415 kDa. Considering the sizes of the poplar GS polypeptides determined by mass spectrometry (Table 1), the resulting values for the GS holoenzymes are compatible with a decameric structure of the enzyme oligomer.

### Catalytic properties of poplar GS isoenzymes

We were interested in determining whether poplar GS holoenzymes differ in their kinetic parameters against substrates because this finding would support their potentially different metabolic roles. The kinetic properties were determined by assaying the biosynthetic GS activity and the most significant results are presented in Table 2. The poplar isoforms did not differ in their affinity for ATP but did demonstrate contrasting kinetic behaviors for ammonium and glutamate. The GS1.1, GS1.2 and GS1.3 enzymes had responses to changes in the concentrations of glutamate which did not follow hyperbolic saturation. The kinetic data were further analyzed using Lineweaver-Burk and Hill plots which indicated the existence of negative cooperativity (Table 2). In contrast, GS2 demonstrated a typical Michaelis-Menten saturation curve for glutamate with a K_m_ value of 26 mM and sigmoidal kinetics against ammonium with calculated parameters of nH = 1.7; S_0.5_ = 0.3. The kinetic analysis also revealed that the poplar cytosolic isoforms of GS exhibited a high affinity for ammonium, particularly GS1.1 with an extremely low K_m_ value (5 μM). Because the final preparations of the enzymes were homogenous and the molecular masses were previously determined (Table 1), it was possible to determine the corresponding catalytic (K_cat_) and specificity constants (K_cat_/K_m_) for ammonium and are also presented in Table 2. The specificity constant for GS1.1 (1.2 × 10^7^ M^−1^ s^−1^) is within a range that is typical of a very efficient catalytic and specific enzyme [17]. In a previous paper [16] we proposed that duplicated genes in poplar may play redundant roles in nitrogen metabolism. If this hypothesis is correct the isoforms encoded by the duplicated genes should have similar metabolic roles, and consequently similar kinetics. To test this hypothesis we recombinantly expressed and characterized the enzymes encoded by the *PtGS1.2* duplicated genes (PtGS1.2-819912; PtGS1.2-716066). The observed kinetic parameters for both expression products were nearly identical (Table 2).

**Table 2:** **Kinetic parameters of poplar GS recombinant enzymes**

	**Ammonium**	**Glutamate**	**ATP**
GS1.1	*K*_*m*_ = 5 μM	Negative cooperativity	*K*_*m*_ = 1.4 mM
		nH = 0.6	
	*K*_*cat/*_ *K*_*m*_ = 1.2 x10^7 a^		
GS1.2			
819912	*K*_*m*_ = 200 mM	Negative cooperativity	*K*_*m*_ = 0.9 mM
716066	*K*_*m*_ = 190 mM	nH = 0.6	*K*_*m*_ = 1.0 mM
	*K*_*cat/*_ *K*_*m*_ = 0.8 x10^6 a^		
GS1.3	*K*_*m*_ = 110 μM	Negative cooperativity	*K*_*m*_ = 1.0 mM
		nH = 0.6	
	*K*_*cat/*_ *K*_*m*_ = 1.1 x10^6 a^		
GS2	Positive cooperativity	*K*_*m*_ = 26 mM	*K*_*m*_ = 0.7 mM
	*nH* = 1.7; *S*_*0.5*_ = 0.3		

The biosynthetic assay was used [39,40].

^a^M^−1^ s^−1^.

### Optimal temperature and pH of poplar GS enzymes

**Figure 3:**
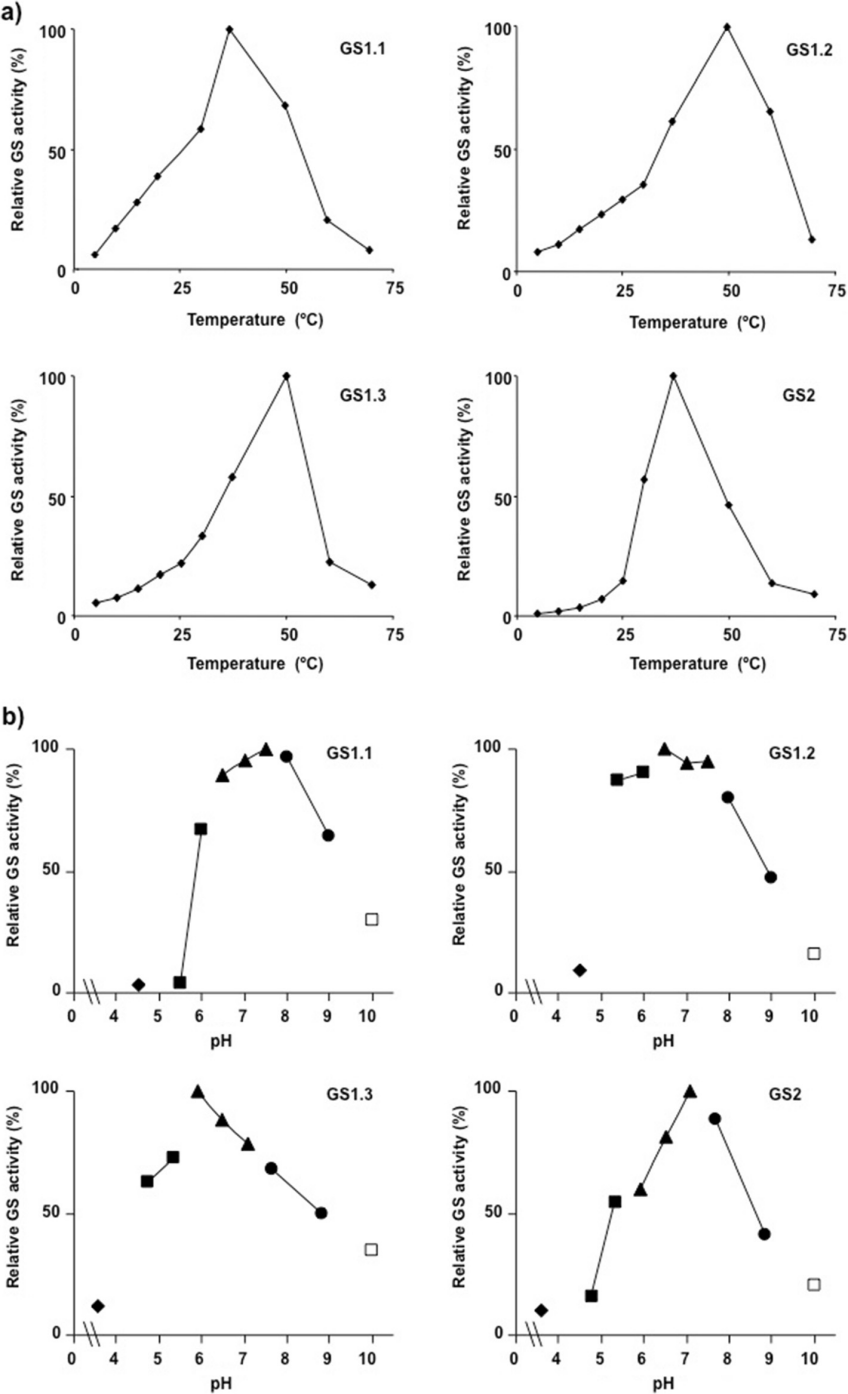
**Effect of temperature and pH in the activity of poplar GS holoenzymes. a)** The activity of purified native isoenzymes were determined at different temperatures from 10°C to 70°C. The maximum level (100%) of activities for GS1.1, GS1.2, GS1.3 and GS2 were 23 nkatal, 65 nkatal, 50 nkatal and 34 nkatal, respectively. Values are the mean ± SD of at least three independent determinations. **b)** The activity of purified native isoenzymes were determined at different pH values. The maximum level (100%) of activity for GS1.1, GS1.2, GS1.3 and GS2 were 38 nkatal, 56 nkatal, 48 nkatal and 25 nkatal, respectively. The following buffers were used: ◆ Acetate (4.5 ), ∎ Mes (5.5-6.5 ), ▲ MOPS (6.6-8), ● Tris (8–9), □ Sodium carbonate (10). Values are the mean ± SD of at least three independent determinations.

The effect of temperature on the activity of the recombinant GS isoforms was examined (Figure 3a). The profiles of the three cytosolic isoforms were quite similar with sustained increases in enzyme activity in response to increases in temperature until a maximum level was reached. However, the profile for GS2 did not demonstrate similar sustained increases in GS activity. The following activation energies were calculated for the cytosolic and chloroplastic GS isoforms: −57.9 kJ mol^−1^ for GS1.1, −41.9 kJ mol^−1^ for GS1.2, −50.4 kJ mol^−1^ for GS1.3 and −107.4 kJ mol^−1^ for GS2 (Additional file 3: Figure S1). Consistent with the observed profiles, similar values were found for GS1.1, GS1.2 and GS1.3, and GS2 exhibited much higher activation energy. GS1.2 and GS1.3 demonstrated maximal activity at 50°C. In contrast, the optimal temperature for GS1.1 was 37°C, which is similar to the observed value for GS2. Poplar GS isoforms were active at a wide range of pH levels, from 5.0 to 9.0 (Figure 3b). The cytosolic enzymes GS1.2 and GS1.3 demonstrated maximal activity at a pH 6.0-6.5 In contrast, the optimal pH for GS1.1 and the GS2 was 7.5.

### Stability of poplar GS isoforms

**Figure 4:**
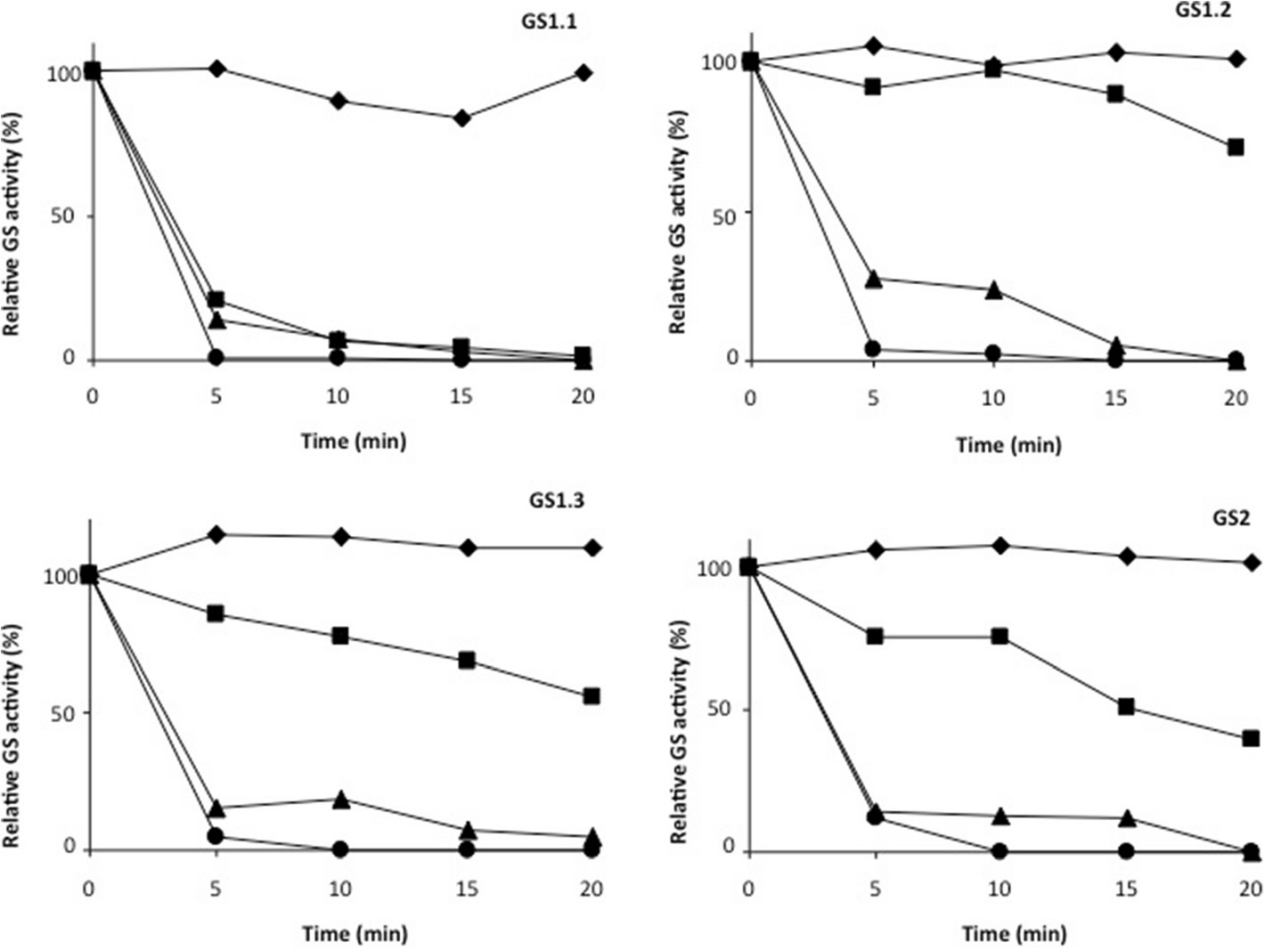
**Thermal stability of poplar GS holoenzymes.** Samples (50 μg) of purified GS2, GS1.1, GS1.2 and GS1.3 native isoenzymes were incubated at different temperatures: ◆ 37°C; ∎ 42°C; ▲ 50°C; ● 60°C. At the indicated periods of incubation (0, 5, 10, 15 and 20 min) samples were removed from the bath and stored on ice until GS activity was determined. The maximum level (100%) of activity for GS1.1, GS1.2, GS1.3 and GS2 was 38 nkatal, 56nkatal, 48 nkatal and 25 nkatal, respectively. Values are the mean ± SD of at least three independent determinations.

To further examine the differences in the poplar isoforms as molecular catalysts the heat stabilities of GS1.1, GS1.2, GS1.3 and GS2 were examined (Figure 4). All enzymes were stable at 37°C, the temperature of the enzymatic assay. The cytosolic enzymes GS1.2 and GS1.3 exhibited a certain degree of thermal stability. They retained more than 50% of their activity when incubated at 42°C and were immediately inactivated at higher temperatures. In contrast, GS1.1 was extremely sensitive to heat, even more sensitive than GS2, and retained only 20% of its activity after 5 min of incubation at 42°C.

Oxidation catalyzed by metals can be used as an indicator of the structural stability of GS enzymes [18]. Therefore, we further examined the tolerance of poplar GS isoforms to metal-mixed oxidation (Additional file 4: Figure S2). As observed for temperature stability, GS1.1 was completely inactived after 180 min of metal-oxidation exposure. In contrast, GS1.2 and GS1.3 were much more tolerant and retained more than 50% of their initial activity level after 240 min of treatment. In this context, it is interesting that the chloroplastic isoform (GS2) was also sensitive to metal-oxidation (Additional file 4: Figure S2).

### Localization of poplar *GS* transcripts in different cell types

**Figure 5:**
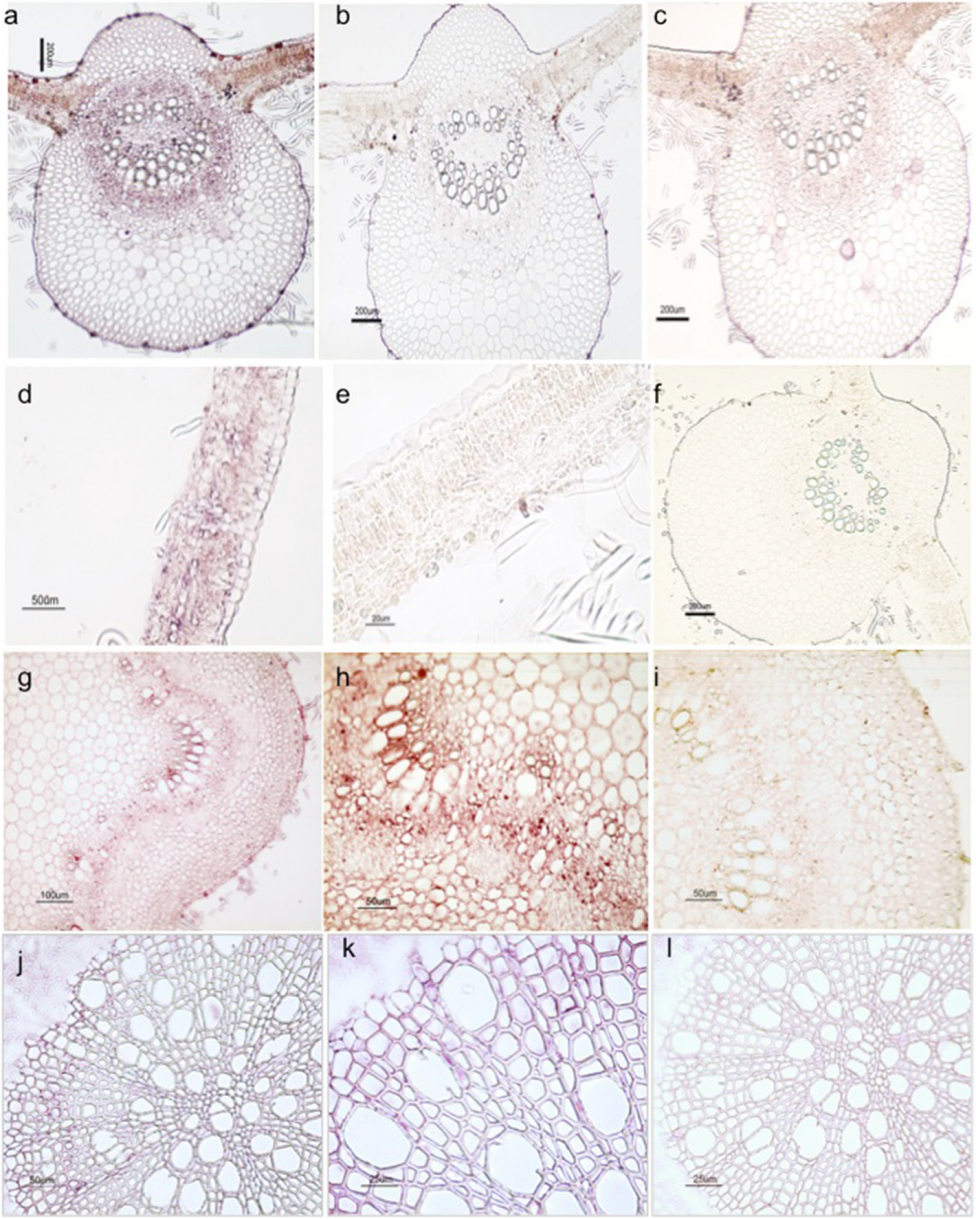
**Cell-type distribution of poplar GS expression analyzed by** ***in situ*** **hybridization.** Cross-sections (10 mm thick) of different poplar organs were subjected to *in situ* hybridization analysis using RNA probes. Leaves: *PtGS2* **(a, d)** and *PtGS1.1* **(c)** antisense; *PtGS2* **(b, e)** and *PtGS1.1* **(f)** sense. Stem: *PtGS1.3* **(g, h)** antisense; *PtGS1.3* **(i)**, sense. Root: *PtGS1.2* **(j, k)** antisense; *PtGS1.2* **(l)** sense probe.

The precise distribution of the *GS* transcripts in the different cell types of the leaves, stems and roots of poplar was examined by *in situ* hybridization (ISH) using specific probes (Figure 5). The specific labeling for *PtGS2* transcripts was observed in the lamina, external phloem and parenchyma cells of leaves (Figure 5a). A magnified view of a leaf blade hybridized with the antisense probe reveals that *PtGS2* mRNA is localized in spongy and palisade cells and that there is a lack of labeling in the lower and upper epidermis (Figure 5d). A similar expression pattern was also observed for the *PtGS1.1* transcripts with enhanced signals in the leaf blade (Figure 5c). *PtGS1.3* mRNA was highly abundant in the vascular bundles of stems (Figure 5g). A strong labeling was observed in phloem cells (Figure 5h). *PtGS1.2* transcripts were localized in the cells of the root vascular cylinder (Figure 5j and k). The specificity of the ISH was confirmed by an absence of signal in the target tissues probed with the sense probes for *PtGS2* (Figure 5b and e), *PtGS1.1* (Figure 5f), *PtGS1.3* (Figure 5i) and *PtGS1.2* (Figure 5l). In a previous paper [16] we proposed that duplicated genes expressed in the same cell types of poplar may play redundant roles in nitrogen metabolism. To test this hypothesis, we performed simultaneous ISH analyses of the two duplicate *PtGS1.3* genes (*PtGS1.3-834185* and *PtGS1.3-827781*), and the results obtained revealed that these genes displayed identical spatial expression patterns.

**Figure 6:**
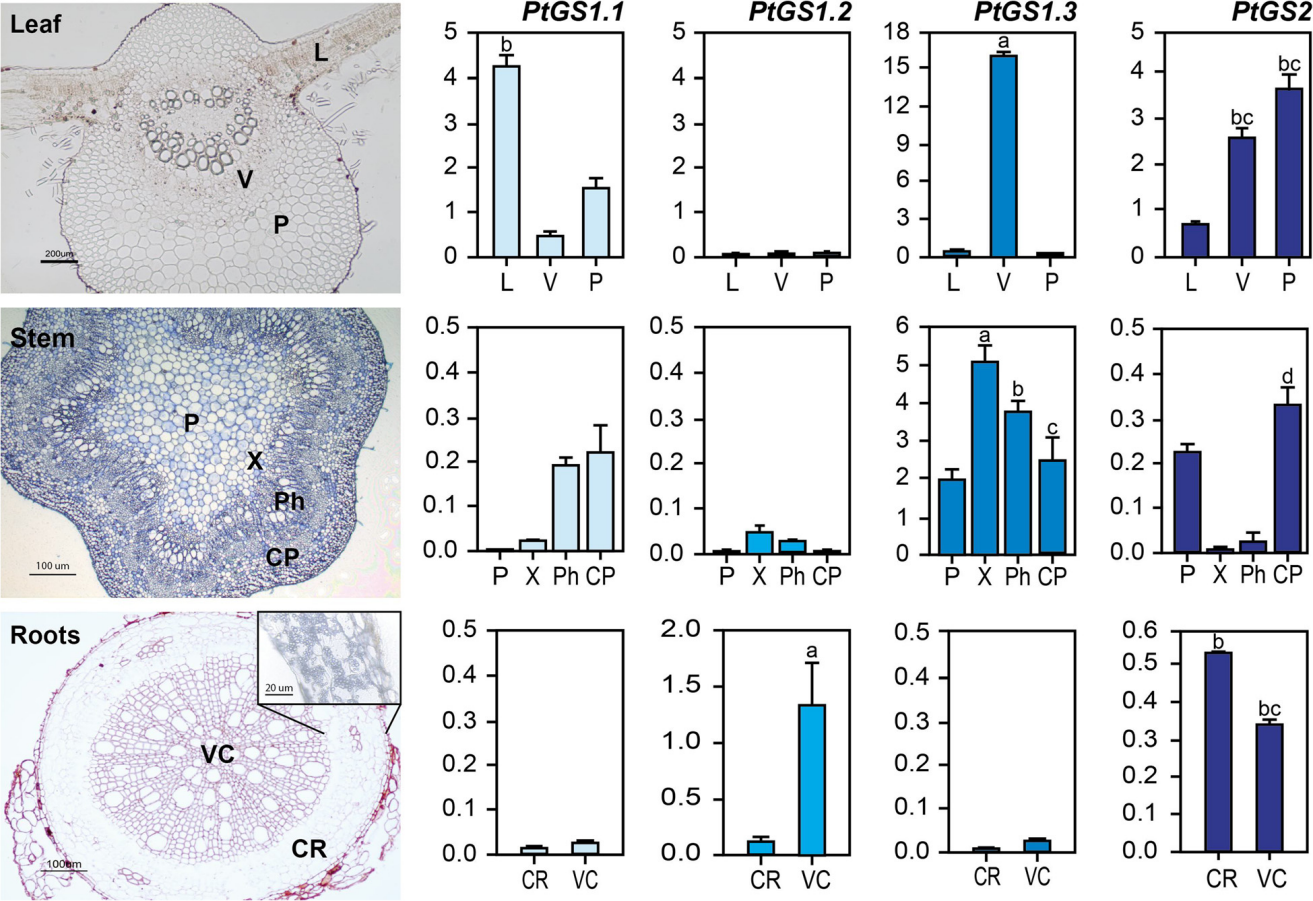
**Cell and tissue distribution of poplar GS expression examined by LCM and qPCR analysis.** Tissue sections from poplar leaves, stems and roots were processed for LCM and total RNA isolated as described in Methods. Three independent biological replicates were processed. The expression levels of duplicated genes *PtGS2*, *PtGS1.1*, *PtGS1.2* and *PtGS1.3* were determined by qPCR using oligonucleotide primers designed to amplify the transcripts encoded by each pair of genes [16]. Leaf: L, Lamina; V, vascular cells; P, parenchyma. Stem: P, pith; X, xylem; F, phloem; CP cortical parenchyma, Root: CR, cortical root; VC, vascular cylinder. Higher magnification shows the presence of numerous plastids in the cortical cells. Each value represents the mean ± SD of 3 biological replicates. Statistics analysis were performed by Anova and the significative diferences were calculated by Tukey’s t test (p < 0.01).

ISH is a powerful technique for studying the spatial expression patterns of genes in plants but the ability to reliably quantify gene expression levels by ISH is quite limited. We were interested in comparing the relative expression levels of each pair of duplicated poplar genes in the specific cell types to complement the results from the ISH analyses and to investigate the roles of the GS family members in poplar ammonium assimilation. To overcome this limitation, we have developed protocols for the Laser Capture Microdissection (LCM) of poplar tissue sections. Total RNA was isolated from LCM samples, and the expression levels of the entire GS gene family were analyzed by real-time qPCR (Figure 6). The maximum level of *PtGS1.1* expression was observed in the leaf lamina and a decreased level was observed in the parenchyma cells of the leaf (Figure 6, leaf). Transcripts for *PtGS1.1* duplicates were also found at lower abundance in the phloem cells and cortical parenchyma of the stems (Figure 6, stem). *PtGS1.2* transcripts were exclusively detected in the vascular cylinder of the root (Figure 6, root). *PtGS1.3* demonstrated the highest levels of gene expression in the LCM samples taken from the leaf and stem and was only detected at low levels in the root (Figure 6). In the leaf, *PtGS1.3* transcripts were exclusively detected in the vascular bundles, but in the stem, *PtGS1.3* transcripts were abundant in all of the cell types examined, with higher levels in the phloem and xylem (Figure 6, stem). A maximum level of *PtGS2* expression was observed in the parenchyma cells of the leaf with a lower level observed in the vascular bundles (Figure 6, leaf). Decreased levels of *PtGS2* transcripts were also found in the pith and cortical parenchyma of the stems (Figure 6, stems). Similar levels of *PtGS2* transcripts were also detected in the cortical parenchyma of the root, where a high abundance of amyloplasts was evident (Figure 6, root inset). It is interesting that the expression profiles of *PtGS1.1* and *PtGS2* in the leaf were complementary with maximum expression levels of *PtGS1.1* in the lamina and maximum expression levels of *PtGS2* in parenchyma cells*.*

### Nitrogen regulation of poplar GS expression

To further investigate the functional properties of GS1.1 in the poplar leaves the expression patterns of the entire gene family of duplicated genes were examined under conditions of adequate (10 mM) and low (0.3 mM) nitrate availability. As Table 3 shows, *PtGS1.1* was predominantly expressed in both young and mature leaves at low nitrogen. Interestingly, the *PtGS1.1* transcripts were particularly abundant in the young leaves. Under conditions of adequate nitrogen nutrition, *PtGS2* transcripts were the most abundant in young leaves, and *PtGS1.1* was the predominant gene expressed in mature leaves.

**Table 3:** **Regulation of the poplar GS gene family by nitrogen availability**

	**Low nitrogen**		**Adequate nitrogen**	
**Gene**	**Young leaves**	**Mature leaves**	**Young leaves**	**Mature leaves**
**PtGS1.1**	2.71 ± 0.15	0.56 ± 0.05	0.06 ± 0.01	0.38 ± 0.02
**PtGS1.2**	0.19 ± 0.09	0.03 ± 0.00	0.08 ± 0.02	0.05 ± 0.01
**PtGS1.3**	0.82 ± 0.40	0.22 ± 0.02	0.32 ± 0.24	0.18 ± 0.01
**PtGS2**	0.28 ± 0.03	0.10 ± 0.00	1.82 ± 0.21	0.07 ± 0.00

Expression levels of poplar GS genes in young and mature leaves under low (0.3 mM) and adequate (10 mM) supply of nitrate.

## Discussion

The evolution of gene families for enzymes should be considered in the context of the metabolic and regulatory networks of the organism and the environment it inhabits.

In poplar, the GS gene family consists of 3 groups of duplicated genes for GS1 (GS1.1, GS1.2 and GS1.3) and 1 duplicated gene for GS2 [16]. A microsynteny analysis of the genomic regions where the GS genes are located suggested that the origin of the duplicated genes was a whole genome-wide duplication (WGD) event that occurred approximately 65 millions year ago and is still detectable over approximately 92% of the poplar genome [19]. The structure of each pair of *Populus* duplicated GS genes is well conserved in both the coding and regulatory regions, and they demonstrate identical spatial and seasonal gene expression patterns [16].

In the first part of this study, we isolated *Populus trichocarpa* GS FLcDNA, produced recombinant poplar isoenzymes in bacteria, and conducted a comparative analysis of their structural and kinetic properties. The aim was to highlight the physiological roles of each isoenzyme according to the previously determined differential expression profile of the duplicated GS genes. The FLcDNA for GS1.1 (PtGS1.1-710678), GS1.2 (PtGS1.2-819912; PtGS1.2-716066), GS1.3 (PtGS1.3-834185) and GS2 (PtGS2-725763) were expressed in *E. coli* to overproduce recombinant isoenzymes*.* All poplar GS isoforms were active in bacteria, which is consistent with results previously described for other GS holoenzymes [20-22], and the observed specific activity levels varied among the different isoforms, with much higher values for the GS1 isoforms than for GS2 (Figure 1). Note that for GS1.2, both of the duplicated genes were produced in *E. coli* for a detailed evaluation of the molecular and kinetic characteristics of both expression products. This analysis was of particular interest considering that isoforms catalyzing the same metabolic reaction are present in the same cell types.

The amounts of available recombinant proteins were sufficient for estimating the molecular sizes of holoenzymes and accurately determining the molecular masses of the poplar GS polypeptides by mass spectrometry analysis. The resulting values (Table 1) are compatible with a decameric structure of the enzyme oligomer, which was previously reported for the cytosolic GS holoenzymes in maize [4] and Medicago [5]. The recombinant expression of poplar GS genes also provided a good source of unlimited amounts of the isoenzymes for the comparative analysis of their biochemical properties. The cytosolic enzymes GS1.2 and GS1.3 exhibited maximal activity levels at high temperatures (50°C) and slightly acidic pH (6.0-6.5). In contrast, the optimal temperature and pH for GS1.1 activity was much lower and closer to GS2. In regard to stability, the cytosolic enzymes GS1.2 and GS1.3 were much more tolerant to thermal inactivation and metal-catalyzed oxidation than GS1.1. Again, the biochemical behavior of GS1.1 was similar to GS2. Previous studies have reported that cytosolic are generally more stable proteins than chloroplastic isoenzymes [23]. Taken together these results indicate that GS1.1 differs from the other poplar cytosolic GS exhibiting an unusually low conformational stability, which is a molecular feature usually characteristic of chloroplastic isoenzymes. It is tempting to speculate that this finding may be related to the possible roles of GS in different environmental conditions or developmental stages.

To understand the correlation between the molecular characteristics and physiological functions, the catalytic properties of the enzymes were examined. According to the kinetic data (Table 2), the turnover number is much smaller for GS2 than for GS1 isoenzymes, which implies that it has a slower production rate of glutamine. The specific positive cooperativity of GS2 for ammonium, however, demonstrates that this enzyme is able to rapidly respond to changes in the ammonium availability in the plastid. Cytosolic GS exhibited negative cooperativity for glutamate as previously observed for the pine GS1b enzyme [21]. This kinetic behavior serves to insulate an enzyme from the effects of changes in substrate concentration [24]. Consequently, GS1.1, GS1.2 and GS1.3 would provide a constant flux of glutamine independently of fluctuations in the celular levels of glutamate. Overall, poplar cytosolic enzymes exhibited similar kinetic characteristics except in their ammonium affinity. Studies in *Arabidopsis thaliana* have shown that the presence of glutamine at residue 49 and serine at residue 174 is related to the high affinity ammonium properties of two GS1 isozymes and that the presence of lysine and alanine in equivalent positions were found in the low affinity GS1 enzymes [25]. Based on these findings, it has been thought that the presence or absence of these residues in the primary structure of the polypeptides may be indicative of the relative ammonium affinity of GS1. For example, in the barley GS family, HvGS1_3 has been proposed to be a low affinity isozyme because it lacks both of these residues and would therefore require a greater concentration of ammonia for maximal activity compared with HvGS1_1 and HvGS1_2 [26]. Differences have also been suggested in the ammonium affinity properties of the GS1 large family of *Brassica napus* because of the presence of these two polar residues at conserved positions [27]. Poplar GS1.1 exhibits an extremely high affinity for ammonium even though it contains lysine and alanine residues at positions that are equivalent to the positions in *Arabidopsis* isoenzymes. Furthermore, a low-affinity GS1 isoform expressed in *Sorghum* roots has a glutamine residue at position 49 [28]. Based on these results, we conclude that the determinant residues of ammonium affinity vary from one GS1 to another depending on the plants species.

To further understand the function of poplar GS genes, the precise gene expression patterns were determined by *in situ* hybridization and laser-capture microdisection analyses. *PtGS2* transcripts were mainly localized in the lamina and parenchyma cells of leaves and found at much lower level in the vascular bundles (Figure 5a, d and Figure 6, stem), which suggest that it plays an essential role in nitrogen metabolism associated with photosynthetic activity [13,14]. Interestingly, the highest levels of the transcripts for *PtGS1.1* duplicates were also observed in the leaf lamina, with decreased levels observed in the parenchyma cells (Figure 5c and Figure 6, leaf). These results suggest that *PtGS1.1* plays a relevant role in the photosynthetic metabolism of the leaf likely complementing the role of PtGS2. Indeed, *PtGS1.1* transcripts were highly abundant in young and mature leaves with low nitrogen, which suggests that the GS1.1 isoform with a high affinity for ammonium plays an important role under these metabolic conditions. OsGln1;1, the predominant isoform in rice leaves [29], is mainly involved in the remobilization of nitrogen released during senescence. In contrast, the predominant GS1 isoform in poplar leaves, PtGS1.1, does not appear to be involved in senescence. When poplar leaves were infected with the bacterial pathogen *Pseudomonas syringae,* the relative abundance of *PtGS2*, *PtGS1.3,* and especially *PtGS1.1* transcripts decreased considerably, which most likely reflects the impact of pathogen attack. In contrast, the levels of *PtGS1.2* transcripts increased dramatically and were greater than 10 times the levels observed in non-infected leaves (Additional file 5: Figure S3). These results are consistent with the enhanced expression of *PtGS1.2* observed in senescent poplar leaves [16] and suggest an essential role for the GS1.2 isoform in nitrogen remobilization. Under conditions of vegetative growth, however, *PtGS1.2* transcripts were almost exclusively expressed in roots, especially in the secondary roots. *PtGS1.2* transcripts were localized in the cells of the root vascular cylinder (Figure 5j and k, Figure 6, root), suggesting that GS1.2 is the principal isoform involved in the primary assimilation of nitrogen from soil. Transcripts for the *PtGS1.3* duplicates were highly expressed in the vascular bundles of leaves and stems (Figure 5g, h, and Figure 6) but were also present at lower levels in the vascular elements of the roots (Figure 6). This specific localization and the particular abundance of *PtGS1.3* in phloem and xylem cells of the stems suggest that the enzyme plays an essential role in generating glutamine and asparagine for nitrogen transport [14,30] and in the reassimilation of ammonium released in phenylalanine metabolism [31,32].

In a previous study [16], we proposed that duplicated genes in poplar may play redundant roles in the nitrogen metabolism of specific cell-types. The analysis of the recombinant isoenzymes encoded by the duplicates PtGS1.2-716066 and PtGS1.2-819912 revealed that they exhibit nearly identical kinetic parameters. These findings strongly suggest that the poplar GS duplicates encode isoenzymes functionally equivalent. It was also of interest to determine whether the duplicated genes displayed identical gene expression patterns. The *in situ* hybridization analysis of transcripts for the *PtGS1.3* duplicates (*PtGS1.3-834185* and *PtGS1.3-827781*) strongly support that poplar gene duplicates are expressed in the same cell-types. Furthermore, the gene expression studies of the duplicated genes in laser microdissected samples from leaves, stems and roots fully support this hypothesis that is consistent with the presence of conserved regulatory elements in the promoters of each pair of genes [16]. Similar results were recently reported for GS genes in *Brassica napus*, in which most of the homologous duplicated genes displayed similar expression patterns in different tissues [27].

## Conclusions

Taken together, the previously reported gene expression analysis of the entire GS family [16], the molecular and functional analysis of the recombinant GS isoenzymes, and the precise locations of the corresponding mRNA in different cell types reported in this study strongly suggest that the poplar GS isoforms play non-redundant roles in tree biology. Furthermore, all these studies further support the proposal that the expression of the duplicated genes in specific cell types serves to increase the abundance of the enzymes. Therefore, while there is no redundancy in the poplar GS family at the whole plant level, it clearly exists in specific cell types that express the two duplicated genes. The preservation of duplicated genes involved in central pathways may be related to the high enzyme copy number needed to maintain metabolic flux [33]. Consequently, GS gene redundancy may contribute to maintaining the homeostasis of nitrogen metabolism during processes associated with the changes in glutamine use in multiple metabolic pathways.

Genome duplications are usually followed by a massive gene loss in which some of the duplicated genes are retained and evolve to new functions [34]. Alternatively, duplicated genes can remain largely redundant and serve to increase the abundance of encoded proteins, or the redundancy could also be related to enhanced robustness against mutations [34]. Functionally, the redundancy in the poplar GS family that appeared after the last WGD most likely favoured the adaptation of poplar trees to faster growth and new ecological niches. This proposal is supported by the increase in growth observed in transgenic poplars overexpressing constitutively a pine GS gene [35]. Furthermore, enhanced GS expression in poplar resulted in an enhanced efficiency in nitrogen assimilation and stress tolerance [36,37]. All these data indicate that increased levels of GS confer selective metabolic advantages in poplar trees. Whereas massive gene loss occurred in the GS gene family of *Brassica napus* [27] following a whole-genome triplication event after divergence from *Arabidopsis*, all of the duplicated genes were retained in *Populus* after the last WGD, and these paralogous genes conserved their expression profiles with no apparent signs of neofunctionalization.

## Methods

### Plant materials

All experiments in this study were performed using hybrid poplar (*Populus tremula x Populus alba*, clone INRA 717 1-B4), and black cottonwood (*Populus trichocarpa*, clone INRA 101–74) micropropagated in vitro on half-strength Murashige and Skoog medium (MS) as previously described [16]. Rooted shoots were transferred to plant growth chambers in plastic pots containing a potting mix (HM3-Agromálaga, Málaga, Spain) and vermiculite in a 1:1 ratio. Plantelets were grown for 2 months in environmentally controlled chambers under previously described conditions [16]. Plants were regularly supplied with a nutrient solution containing 10 mM potassium nitrate.

### Cloning of poplar GS FLcDNA and insertion into expression vectors

Total RNA from *Populus trichocarpa* leaves was used to generate cDNA [16]. The cDNA obtained was used as a template to obtain coding sequences (CDS) of GS using a PCR strategy. The PCR reaction was conducted using AccuSure DNA polymerase (Bioline, London, United Kindom). The PCR conditions were: 1 cycle: 95°C, 10 min; 35 cycles: 95°C, 30 s; 55°C, 30 s; 72°C, 90 s; 1 cycle: 72°C, 10 min.

The primers were designed according the GS sequences from the *Populus trichocarpa* genome (http://genome.jgi-psf.org/). Numeric identifiers for the CDS are the same previously used for the corresponding genes [16]. The forward primer sequences were redacted beginning with the ATG triplet except for the chloroplastic protein, in which the codon encoding the first common amino acid obtained in an alignment of plant GS2 sequences was used. A restriction site in the 5′ region was then added to these primer sequences (underlined). The reverse primers were also designed to end in the stop codon, except for the GS1.1 in which the penultimate codon was selected. Restriction sequences were then also added in the 5′ antisense regions (underlined).

The PCR products were first subcloned in the SmaI site of the pGEM-3Zf(+) vector using the blunt end strategy except the GS1.2 CDS, which had a forward primer with a previously inserted 5′ PstI site (double underlined) for the insertion of the PCR product into the corresponding site of the vector. The pGEM-3Zf(+) constructs and a pET-28a(+) expression vector (Invitrogene, CA, USA) were then treated with the restriction enzymes to subclone the CDS in this vector. This strategy generated recombinant polypeptides with a poly-His-tag in the N-terminal region, except GS1.1, which harbored the tag in the C-terminal region (Additional file 1: Table S1).

### Real-Time quantitative PCR

The relative quantification of the gene expression was performed exactly as previously described using the primers designed to amplify specifically the transcripts encoded by each pair of duplicated genes [16].

### Overproduction of recombinant enzymes in bacteria

The transformed *E. coli* strain BL21(DE3)-RIL with the pET-28a(+) vectors were grown at 25°C in 3 liters of Luria-Bertani medium supplemented with kanamycin (ml-1) and chloramphenicol (ml-1). When the O.D. of the cultures was 0.4 at 600 nm, the temperature was lowered to 10°C, and then 0.1 mM of Isopropyl-β-D-thiogalactoside (IPTG) was supplied to induce the expression of the recombinant proteins. The cells were incubated for hours until an O.D. value of 0.9 was reached.

### Extraction and purification of recombinant enzymes from bacteria

All operations were carried out at 4°C. Cells were collected by centrifugation (10 min, 4,000 × g) and resuspended (1 g of pellet in 3 mL of buffer A: 25 mM Tris pH 8, 5 mM mercaptoethanol, 1 mM MnCl_2_). The bacteria were lysed by incubation 30 min with 1 mg/mL lysozyme and then sonication with a microprobe emitting 10 pulses of 4 seconds and 10 s intervals, at the intensity level 4 from a Branson sonifier-250 (Branson Ultrasonics, CT, USA). The soluble fraction was cleared by centrifugation (22,000 × g, 30 min).

Proteins were purified on the basis of the His-tag tail. A total 30 mg of total protein from bacterial soluble fraction were loaded on a column prepared with 7.5 mL of protino Ni-IDA resin (Macherin-Nagel, Düren, Germany) equilibrated with buffer A. The column was washed with 20 volumes of bed, and the protein was eluted using a 0–50 mM imidazole gradient in buffer A. The eluted protein was concentrated using Amicon Ultra 0.5 mL centrifugal filters MWCO 10 kDa from Millipore corporation (Maryland, MD, USA), and the final preparations were stored in 30% glycerol in buffer A at 4°C. Protein concentration was determined using the Bradford’s procedure [38]. Immunoblots were performed as described elsewhere [39].

### Determination of enzyme activity

GS activity was determined using the synthetase and biosynthetic assays [39,40]. Different buffers were used instead when determining the GS activity at different pH levels.

### Gel filtration chromatography

Purified proteins were loaded on a Sephacryl S-300 gel filtration column (100 cm × 1.8 cm) equilibrated in buffer A using a flux of 10 ml/h. The column was calibrated with molecular weight protein standards (Gel Filtration Markers Kit MWGF1000 Sigma-Aldrich St. Louis, MO, USA). The fractions were collected and GS elution was determined by the synthetase assay.

### Metal catalyzed oxidation assays

Samples used for metal oxidation analysis were fractions with GS activity collected from ionic exchange chromatography, concentrated with ammonium sulfate, and dialyzed three times in buffer A (without Mn^2+^) for a total of 6 h. The incubations were carried out in a final volume of 1 ml for 6 h at 4°C. The incubated samples contained the sample, 15 mM ascorbate, and 0.2 mM FeCl_3_.

### Mass-spectrometry analysis

Purified recombinants proteins (100 ng/μL) were loaded onto the MALDI plate followed by 1 μL of the alpha-cyano-4-hydroxycinnamic acid matrix (5 mg/mL in ACN/TFA 0.2%, 1:1); acetonitrile LS-MS CHROMASOLV (ACN) and trifluoroacetic acid were purchased from FLUKA (Sigma-Aldrich, St. Louis, MO, USA). The MS analyses were conducted in a 4700 Proteomics Analyzer mass spectrometer (ABSCIEX, Foster City, CA, USA) working in the linear positive ion mode at 20 kV Source 1 acceleration voltage. The Grid 1 voltage was set to 92.5% of the acceleration voltage. The delay time was 850 ns, the low mass gate was enabled with an offset of 0.0 and data were accumulated between 2000 and 60000 Da. Each data point was the summation of 20 spectra, acquired with 50 laser shots.

### *In situ* hybridization

Stem and leaf tissues of poplar plants growing in growth chambers were fixed in 4% formaldehyde and 0.25% glutaraldehyde for 3 h at room temperature. Plant tissue was vacuum-infiltrated for 15 min once an hour and remained in fixative at 4°C overnight to allow complete substitution. Next, the samples were washed in PBS, dehydrated in a graded ethanol series, gradually infiltrated with paraplast X-TRA® (Sigma-Aldrich) and sectioned (10 μm thick) for *in situ* mRNA localization according to Cantón et al. [41] and Craven-Bartle et al. [32].

A 3′-end, non-coding fragment from a cDNA encoding the *P. trichocarpa* isoforms GS were subcloned into the pGEM®^−^3Zf (+) vector (Promega) and this construct was used for synthesis of digoxigenin-labeled antisense and sense RNA probes using the DIG RNA Labelling mix (Roche). The probes were purified with the NucleoSpin® RNA Clean-Up XS kit (Macherey-Nagel). The DIG-labeled RNA probe yields were estimated by comparing the intensity of the sample to the defined control made with DIG-labeled control RNA (Roche). Hybridization was conducted at 55°C over night. Probe bound to the section was detected using anti-digoxigenin Fab conjugated with alkaline phosphatase and NBT/BCIP as chromogenic substrates (Roche). Brightfield images were captured using an Eclipse E-800 microscope (Nikon, Kingston upon Thames, UK).

### Laser capture microdissection (LCM)

Two-month-old plantelets of hybrid poplar (*Populus tremula x Populus alba)* were sampled and 0.5 cm tissue sections were processed for LCM. The leaf and stem sections were fixed with acetone and paraffin embedded. The root sections were mounted in a specimen holder with embedding medium Tissue-Tek optimal cutting temperature (OCT) (Sakura Finetek, The Netherland) and snap-frozen in liquid nitrogen for cryostat sectioning.

The paraffin embedded samples were fixed in acetone by freeze substitution at −80°C during 3 weeks, and then were tempered to 4°C o/n. The acetone was then sequentially replaced with acetone for 1 h, acetone:Histolemon (1:1) for 1 h, pure Histolemon (Carlo Erba, Milan, Italy) for 1 h, and then 5 to 6 pearls of Paraplast X-tra (Leica Microsystems, Wetzlar, Germany) were add to the Histolemon and incubated for 1 h at RT. Later, an equal volume of molten Paraplast X-tra was added to the samples at 58°C and incubated for 2 h. Finally the Histolemon:Paraplast X-tra mix was replaced by pure liquid Paraplast X-tra at 58°C. The liquid Paraplast X-tra was replaced 4 times during one day before forming the blocks. The embedded samples were stored at 4°C before sectioning. The samples were cut with a rotary microtome and the sections (10 μm thick) were mounted on PET-membrane 1.4 μm steel frames (Leica Microsystems, Wetzlar, Germany) and dried for 1–2 h at 37°C. Dry slides were deparaffinized twice in Histolemon for 5 min each. Subsequently the samples were incubated in ethanol 100% for 5 min, and air dried for 5 min. Laser microdissection was performed with a LMD700 instrument (Leica, Germany).

The root samples were embedded in OCT medium, snap-frozen in liquid nitrogen and stored at −80°C. One day before the cryostat-sectioning, the samples were tempered at −20°C. Fourteen μm thick sections were made with a Thermo Scientific HM 525 Cryostat (VWR International, PA, USA) at −20°C, and mounted on PET-membrane 1.4 μm steel frames using a Plexiglass frame Support (Leica, Germany). The steel frames containing the samples were used immediately or stored at −80°C until use. Prior to the microdissection operations, the samples were fixed in cold ethanol 100% for 10 sec, deprived of OCT medium with DEPC treated water for 2 minutes, and refixed in ethanol 100% for 1 minute. Subsequently the samples were air dried and microdissected with a LMD700 instrument.

The microdissected samples were placed into the caps of 0.5 mL tubes containing 10 μL of lysis buffer from an RNAqueous-Micro RNA Isolation Kit (Ambion, TX, USA). These samples could be stored at −80°C to use later. The RNA was obtained with the same Kit. LCM protocol of the kit was followed for the paraffin-embedded samples, and non-LCM protocol for the snap-frozen samples. RNA quality was assessed using the RNA Pico Assay for the 2100 Bioanalyzer (Agilent, CA, USA).

### Availability of supporting data

All the supporting data of this article are included as additional files.

## Additional files

Additional file 1:
**Nucleotide sequences of the full-length cDNA encoding members of the poplar GS family.**
Additional file 2:
**Table S1.** Poplar GS accession numbers, primers and restriction treatments for poplar GS cloning. Additional file 3:
**Figure S1.** Values of activation energy of poplar GS holoenzymes. The activation energy (Ea) for each recombinant GS was calculated from the slope of the Arrhenius plots. Additional file 4:
**Figure S2.** Effect of metal-catalyzed oxidation on poplar GS holoenzymes. ◆: 0 mM FeCl_3_ . ∎: 1 mM FeCl_3_. Additional file 5:
**Figure S3.** GS transcript levels in poplar leaves infected with the pathogen *Pseudomonas syringae*. Each value represents the mean ± SD of 3 biological replicates. Statistics analysis were performed by Anova and the significative diferences were calculated by Tukey’s t test (p < 0.01). □ Non-infected ∎: Infected.

## Abbreviations

CDS: coding sequence
GS: glutamine synthetase
GS1: cytosolic glutamine synthetase
GS2: chloroplastic glutamine synthetase
GOGAT: glutamate synthase
IPTG: isopropyl-β-D-thiogalactoside
MS/MALDI: mass spectrometry/ matrix-assisted laser desorption/ionization
LCM: laser capture microdissection
OCT: optimal cutting temperature
qPCR: real-time quantitative PCR
WGD: whole genome-wide
