# Supplementary material for: Redundancy and metabolic function of the glutamine synthetase gene family in poplar
Source: BMC Plant Biol. 2015 Jan 22;15:20. doi: 10.1186/s12870-014-0365-5 (PMC4329200; doi:10.1186/s12870-014-0365-5)
Supplement: Additional file 2: — Table S1. Poplar GS accession numbers, primers and restriction treatments for poplar GS cloning. [file 12870_2014_365_MOESM2_ESM.docx]

| **GS CDS clones** | **Forward primer** | **Reverse primer** | **Restriction treatment of the PCR product subcloned in the pGEM-3Zf(+) vector** | **Restriction treatment of the pET-28a(+) vector** |
| --- | --- | --- | --- | --- |
| PtGS2-725763 | CCATTAATTTTGCCCTCAAGTCTGAG | GGGAATTCTCAGACATTCATAGACAGC | Ase I (5´) and EcoRI (3´) | NdeI (5´) and EcoRI (3´) |
| PtGS1.1-710678 | CCCACATGTCGTTGCTTTCAGACCTT | GGGCTCGAGTGGCTTCCACAGGATGGTGG | PciI (5´) and XhoI (3´) | NcoI (5´) and XhoI (3´) |
| PtGS1.2-819912 | CCCTGCAGATTAATATGTCTCTCCTT | GGGAATTCCTATGGCTTCCAGATAAT | Ase I (5´) and EcoRI (3´) | NdeI (5´) and EcoRI (3´) |
| PtGS1.2-716066 | CCCTGCAGATTAATATGTCTCTCCTT | GGGTCGACCTATGGCCTCCAGATAAT | Pst I (5´) and SalI (3´) | NdeI (5´) and SalI (3´) |
| PtGS1.3-834185 | CCATTAATATGTCTCTCTTGTCTGAT | GGGTCGACACTTATGGCTTCCAGAGA | AseI (5´) and SalI (3´) | NdeI (5´) and SalI (3´). |
