# Supplementary figures and images for: Redundancy and metabolic function of the glutamine synthetase gene family in poplar
Source: BMC Plant Biol. 2015 Jan 22;15:20. doi: 10.1186/s12870-014-0365-5 (PMC4329200; doi:10.1186/s12870-014-0365-5)

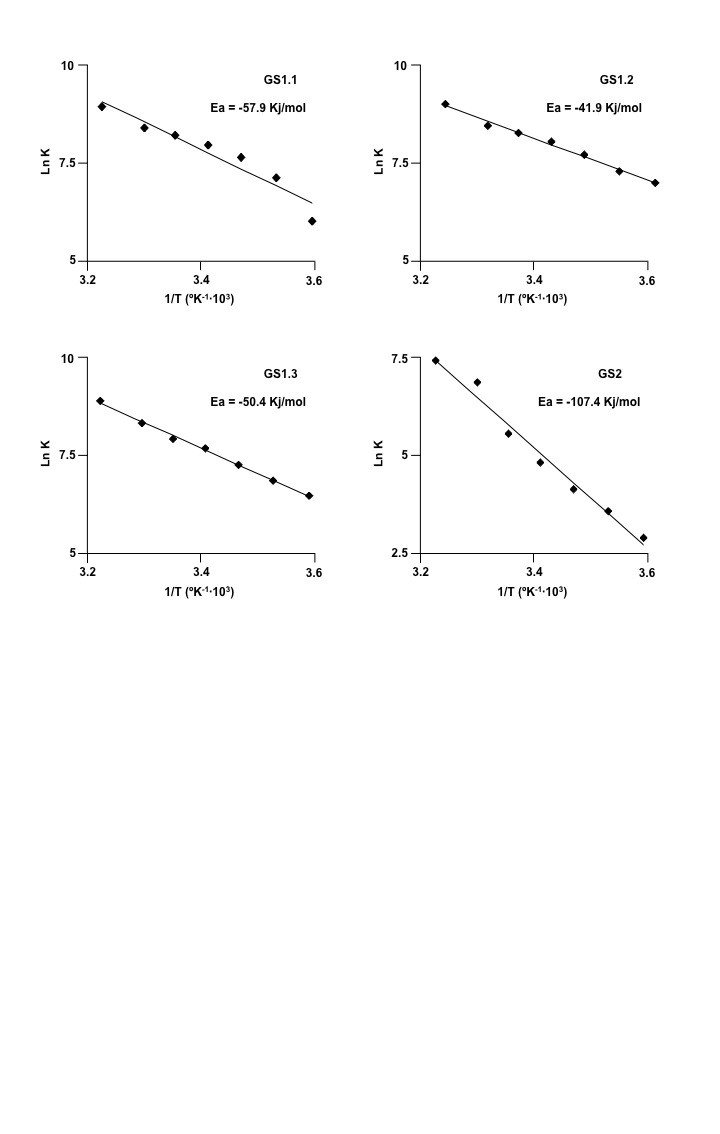

Supplement: Additional file 3: — Figure S1. Values of activation energy of poplar GS holoenzymes. The activation energy (Ea) for each recombinant GS was calculated from the slope of the Arrhenius plots. [file 12870_2014_365_MOESM3_ESM.jpeg]

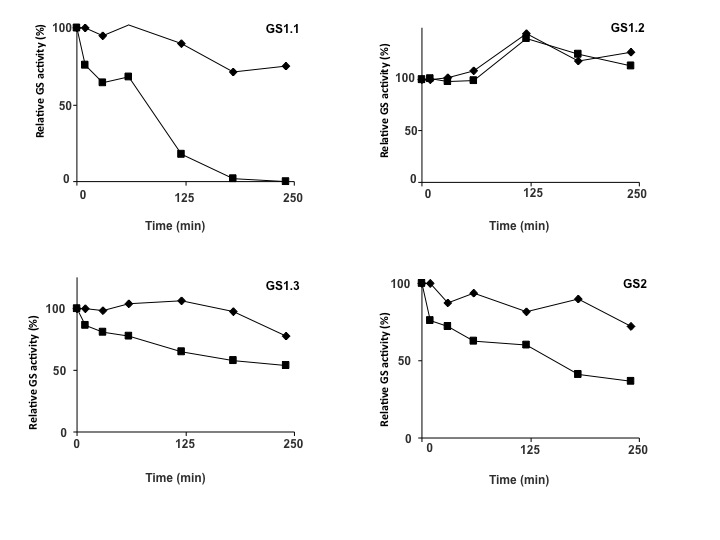

Supplement: Additional file 4: — Figure S2. Effect of metal-catalyzed oxidation on poplar GS holoenzymes. ◆: 0 mM FeCl3 . ∎: 1 mM FeCl3. [file 12870_2014_365_MOESM4_ESM.jpeg]

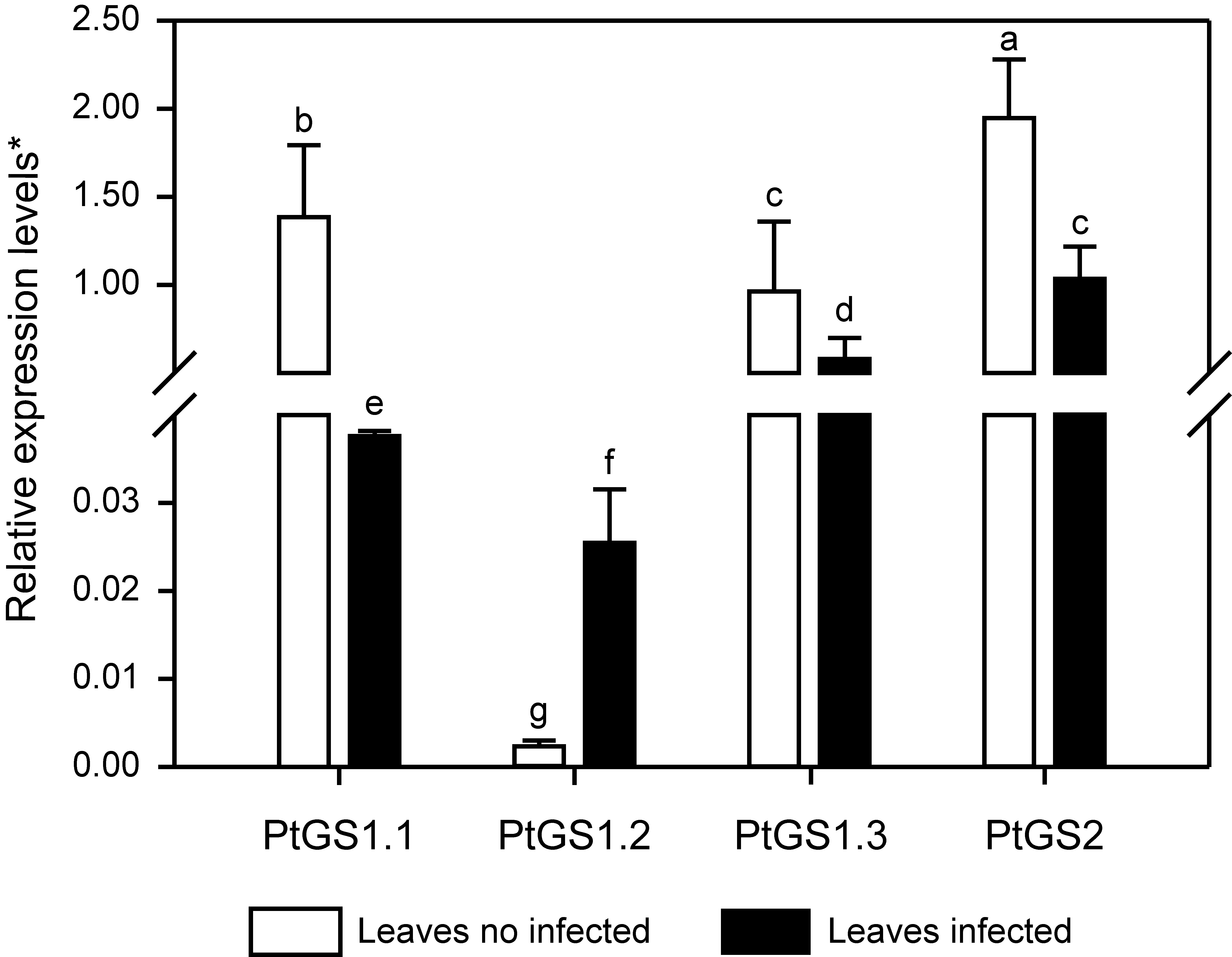

Supplement: Additional file 5: — Figure S3. GS transcript levels in poplar leaves infected with the pathogen Pseudomonas syringae. Each value represents the mean ± SD of 3 biological replicates. Statistics analysis were performed by Anova and the significative diferences were calculated by Tukey’s t test (p < 0.01). □ Non-infected ∎: Infected. [file 12870_2014_365_MOESM5_ESM.jpeg]
